# Experience of gratitude, awe and beauty in life among patients with multiple sclerosis and psychiatric disorders

**DOI:** 10.1186/1477-7525-12-63

**Published:** 2014-04-30

**Authors:** Arndt Büssing, Anne Gritli Wirth, Franz Reiser, Anne Zahn, Knut Humbroich, Kathrin Gerbershagen, Sebastian Schimrigk, Michael Haupts, Niels Christian Hvidt, Klaus Baumann

**Affiliations:** 1 Quality of Life, Spirituality and Coping, Institute of Integrative Medicine, Faculty of Health, University Witten/Herdecke, Herdecke, Germany; 2 Senior Research Fellow Freiburg Institute for Advanced Studies (FRIAS), University of Freiburg, Freiburg, Germany; 3 Caritas Science and Christian Social Work, Faculty of Theology, University of Freiburg, Freiburg, Germany; 4 Department of Psychiatry and Psychotherapy, University Clinic Freiburg, Freiburg, Germany; 5 Department of Neurology, Communal Hospital Herdecke, Herdecke, Germany; 6 Department of Neurology and Palliative Care, Köln-Merheim Hospital, Cologne, Germany; 7 Neurological Hospital, Clinic of Lüdenscheid, Lüdenscheid, Germany; 8 Augustahospital Anholt, Neurological Hospital, Isselburg -Anholt, Germany; 9 Faculty of Health Sciences, University of Southern Denmark, Odense, Denmark; 10 Clinic and Polyclinic for Palliative Medicine, Ludwig Maximilian University, Munich, Germany

**Keywords:** Gratitude, Awe, Spirituality, Life satisfaction, Multiple sclerosis, Psychiatric disorders

## Abstract

**Background:**

Feelings of gratitude and awe facilitate perceptions and cognitions that go beyond the focus of illness and include positive aspects of one’s personal and interpersonal reality, even in the face of disease. We intended to measure feelings of gratitude, awe, and experiences of beauty in life among patients with multiple sclerosis and psychiatric disorders, particularly with respect to their engagement in specific spiritual/religious practices and their life satisfaction.

**Methods:**

We conducted a cross-sectional survey with standardized questionnaires to measure engagement in various spiritual practices (SpREUK-P) and their relation to experiences of *Gratitude, Awe and Beauty in Life* and life satisfaction (BMLSS-10). In total, 461 individuals (41 ± 13 years; 68% women) with multiple sclerosis (46%) and depressive (22%) or other psychiatric disorders (32%) participated.

**Results:**

Among participants, 23% never, 43% rarely, 24% often, and 10% frequently experienced *Gratitude*. In contrast, 41% never, 37% rarely, 17% often, and 6% frequently experienced *Awe. Beauty in Life* was never experienced by 8% of the sample, and 28% rarely, 46% often, and 18% frequently experienced it. *Gratitude* (F = 9.2; p = .003) and *Beauty in Life* (F = 6.0; p = .015) were experienced significantly more often by women than men. However, the experience of *Awe* did not differ between women and men (F = 2.2; n.s.). In contrast to our hypothesis, *Gratitude/Awe* cannot explain any relevant variance in patients’ life satisfaction (R^2^ = .04). Regression analyses (R^2^ = .42) revealed that *Gratitude/Awe* can be predicted best by a person’s engagement in religious practices, followed by other forms of spiritual practices and life satisfaction. Female gender was a weak predictor and underlying disease showed no effect.

**Conclusions:**

*Gratitude/Awe* could be regarded as a life orientation towards noticing and appreciating the positive in life - despite the symptoms of disease. Positive spirituality/religiosity seems to be a source of gratitude and appreciation in life, whereas patients with neither spiritual nor religious sentiments (R-S-) seem to have a lower awareness for these feelings.

## Background

Prior research has focused mainly on the experience of gratitude among patients with fatal, potentially life-threatening diseases (i.e., cancer survivors, HIV infected). Until now, little attention has been given to the perception of gratitude among patients with chronic diseases that are not primarily fatal and life-threatening (i.e., multiple sclerosis and psychiatric disorders). Therefore, we were interested in how patients with chronic, nonterminal illnesses adapt to their situation and experience gratitude, awe, and beauty in life. Do these patients really perceive gratitude and awe despite their disease and its unpredictable course?

Patients with chronic disease are often burdened by their thoughts and feelings and tend to focus on symptoms and relief. Awareness of their thoughts and feelings is often considerably narrowed. Yet, patients may also at the same time become more aware of moments and experiences of (silent) well-being, interactions with others and care, which may lead to favorable feelings of gratitude and joy. Feelings of gratitude facilitate perceptions and cognitions that go beyond the focus of illness and include positive aspects of one’s personal and interpersonal reality, even in the face of disease. These feelings may help those with chronic or other illnesses to enjoy the beauty of nature, their surroundings, relationships with others, as well as the goodness of life. As such, they become a source of relief and even of lasting fulfilment, notwithstanding the limitations of the disease [1]. The key issue here is awareness of such positive experiences and relationships. Hence, it is a matter of attention, of interpretation and of acceptance of the situation in the light of the beauty still found in the middle of a crisis [2].

Gratitude is thought to be an “emotional state and an attitude toward life that is a source of human strength in enhancing one’s personal and relational well-being” [3]. Gratitude in perception, feeling, cognition, motivation, and memory constitutes a counterbalance - or even counterforce - against physical and cognitive limitations of the disease. With regard to the past, gratitude is typically associated with memories of love and joy, both received and given. With regard to the present, gratitude enables the perception of enriching experiences in the midst and in spite of illness. Looking to the future, gratitude reinforces confidence and hope. Undoubtedly, gratitude is an essential dimension of one’s subjective quality of life, patients included [4].

Feelings, cognitions and motivations related to gratitude are intrinsic psychological occurrences – they cannot be forced or commanded. Yet, they can be consciously trained and harbored [4-7]. Feelings of gratitude can be, and clearly are, often thwarted by the immensity of disease and the physical, psychological and spiritual challenges it entails [8].

In contrast, awe is an emotional perception of wondering astonishment and admiration, i.e., when facing either a breathtaking landscape, a starry sky, experiencing mystical experiences, etc. [9,10]. This overwhelming emotion is often accompanied by a sense of vastness and the feeling that time is standing still. Keltner and Haidt [9] suggested that such emotions are experienced more often in “times of tremendous social change”. Patients with chronic, long-lasting, and thus a frustrating course of disease also undergo dramatic social changes. Thus, it is reasonable that awe may also be experienced more frequently during times of illness. The experience of beauty in life is a more general feeling that can but does not always result in feelings of gratitude or awe. It is simply the ability to recognize and perceive such beauty in nature, in beloved persons, etc.

While presumably all religious traditions try to come to grips with suffering, Abrahamic religious traditions underline that humans, nature and all living things stem from God, the loving creator of all that exists. This belief in a loving God can prevail against the negative experience of disease. Spirituality (either religious or non-religious) may facilitate sentiments of gratitude in the midst of an individual’s suffering. Openness to spirituality also opens people to perceive, receive, and respond to the fundamental goodness of creation and life. Such feelings and behaviors of gratitude are a basic expression of religion. Gratitude becomes a part of religious awe in relation to God (or, in other currents, to some transcendent power) both in feeling and prayer and behavior [11-13]. Thus, is appears meaningful to ask whether the religious/spiritual attitudes of patients have an influence on their experience of gratitude and chronic disease. This will be one of the main research questions of this study.

Research on gratitude as a life orientation has shown that gratitude is correlated with positive emotional functioning, lower dysfunction, positive social relationships, and well-being (reviewed in [14]). Interestingly, thankfulness was also associated with lower risk of major depression, generalized anxiety disorder, phobia, and drug abuse [15]. The association between gratitude and fewer depressive symptoms seems to be mediated by positive reframing and/or positive emotion [16].

Gratitude has been examined in the face of various chronic diseases, including cancer [17-21] and HIV infection [22]. Persons with prostate cancer sought to appreciate the “beauty and gifts of life” [17]. Long-term breast cancer survivors expressed their “deep gratitude for being alive”; they reported “joy for life itself and for being present in their lives” and a “more positive approach to life” [18]. The authors suggested that “this gratitude helped the women balance the challenges and discomforts; many of them endured as long-term survivors” [18].

Due to the adaptive role of positive emotions under chronic stress, Algoe and Stanton [21] suggested that gratitude could be regarded as a factor of resilience. In a study involving patients with metastatic breast cancer, feeling gratitude resulted in a readiness to accept help from supporting persons [21]. Among skin cancer patients, however, those with malignant melanoma were more likely to “mention a sense of relief/gratitude following treatment and/or a commitment to enjoy life here on” than patients with non-melanoma skin cancer [19]. Because the “realization of one’s mortality” and emotional relationships were more frequent in melanoma patients than in the non-melanoma skin cancer patients [19], one may suggest that these factors might contribute to the higher proportion of relief/gratitude. Another study investigated cancer patients who experienced posttraumatic growth and found that their positive experience was associated with “cognitive reappraisal of emotion, gratitude finding, and openness to experience” [20].

All of these specific findings were derived from patients with fatal, potentially life-threatening diseases, and gratitude was expressed either as renewed awareness of the gift of life (diagnosis and treatment), or as an appreciation of still being alive (survivors). However, what about patients with chronic diseases that are not primarily fatal and life-threatening, with symptoms that may appear “at random” or that may persist for longer periods of time? Will the experience of gratitude and awe differ with respect to specific forms of diseases?

To answer these questions, we intended to measure feelings of gratitude and awe, along with the experience of beauty in life in two rather diverse patient groups: one group with multiple sclerosis (MS) and another group with psychiatric disorders. We were particularly interested in associations between these variables and spiritual attitudes and life satisfaction. Patients with the aforementioned diseases were chosen (1) because one can expect a large fraction of relatively young individuals who have low specific interest in spiritual or religious issues [23-25] (this provides a basis for comparison to those who do regard themselves as religious and/or spiritual), and (2) because they experience chronic diseases that are not primarily fatal and life-threatening. Patients with MS are faced with an illness that is characterized by an often unpredictable course of exacerbations and remissions with significant impairment of life goals and everyday life. Furthermore, there is no “cure”. Similarly, patients with psychiatric disorders cannot expect a specific “cure” in most cases, and thus they must also find strategies to adapt to their recurring symptoms.

For the purpose of this study, we therefore measured whether and how often these patients have experienced feelings of gratitude, wondering awe, and beauty in life. To measure these emotions and perceptions, we used specific items derived from a questionnaire for measuring the frequency of engagement in specific religious and secular forms of spirituality [23], particularly the scale *Gratitude/Awe,* which includes three items addressing the frequency of feelings of great gratitude, wondering awe, and experienced and valued beauty in life. We suggest that (1) the frequency of experience will differ with respect to specific forms of spirituality, particularly that religious/spiritual patients may experience gratitude, awe and beauty in life more often than non-religious/skeptical patients, (2) that the perceptions of these emotions differ with respect to gender and underlying disease, and (3) that these emotions are related to patients’ life satisfaction.

## Methods

This cross-sectional survey among individuals with multiple sclerosis and psychiatric disorders (Table 1) used standardized questionnaires which are described in detail in the following paragraphs.

**Table 1:** Sociodemographic and psychometric characteristics of 461 patients

**Variables**	**Entire sample (100%)**	**Multiple sclerosis**^**§ **^**(46%)**	**Depressive disorders (32%)**	Psychotic and other disorders (22%)*	**Statistics (Pearson’s χ**^**2 **^**or ANOVA)**
**Gender, %**					p < .0001
Women	68	78	66	56	
Men	32	22	34	44	
**Age, years** (Mean, standard deviation)	41 ± 13	43 ± 11	41 ± 14	38 ± 13	F = 5.2; p = .006
**Family status,** %					p < .0001
Married	58	56	50	67	
With partner, not married	22	17	34	21	
Divorced	11	19	0	0	
Single	10	8	16	12	
**Educational level,** %					p = .007
Through Grade 9	24	22	26	26	
Through Grade 10	33	33	37	31	
High school diploma	38	36	37	41	
Other	5	9	0	1	
**Spiritual/religious self-categorization,** %					n.s.
R + S + (religious and spiritual)	15	12	16	18	
R + S- (religious, not spiritual)	21	19	24	21	
R-S + (not religious but spiritual)	15	16	14	14	
R-S- (neither religious nor spiritual)	50	54	47	46	
**Engagement in spiritual activities** (0–100)**					
Existential practices	54 ± 25	43 ± 11	60 ± 23	62 ± 22	F = 23.2; p < .0001
Humanistic practices	65 ± 19	65 ± 19	66 ± 16	65 ± 20	F = 0.1; n.s.
Religious practices	26 ± 24	22 ± 24	31 ± 25	27 ± 23	F = 5.1; p = .007
Spiritual (MB) practices	19 ± 22	21 ± 24	15 ± 17	19 ± 21	F = 2.6; n.s.
Gratitude/Awe	42 ± 24	44 ± 22	38 ± 25	43 ± 25	F = 2.2; n.s.
**Life satisfaction Sum Score (BMLSS-10)** (0-100)	54.6 ± 22.3	69.1 ± 17.1	42.5 ± 15.6	42.5 ± 20.7	F = 123.1; p < .0001
**Life Satisfaction Subdimensions** (0–6)					
Family life	3.62 ± 1.83	4.60 ± 1.45	2.83 ± 1.55	2.84 ± 1.84	F = 66.0; p < .0001
Friendships	3.70 ± 1.73	4.49 ± 1.43	3.03 ± 1.64	3.06 ± 1.73	F = 46.7; p < .0001
Work situation	2.82 ± 1.96	3.69 ± 1.72	2.30 ± 1.75	2.18 ± 1.98	F = 28.2; p < .0001
Myself	2.81 ± 1.80	4.00 ± 1.40	1.82 ± 1.44	1.88 ± 1.53	F = 121.5; p < .0001
Where I live	4.01 ± 1.64	4.55 ± 1.42	3.50 ± 1.61	3.62 ± 1.71	F = 22.1; p < .0001
Life in general	3.32 ± 1.69	4.39 ± 1.20	2.21 ± 1.37	2.32 ± 1.66	F = 142.6; p < .0001
Financial situation	3.24 ± 1.83	3.93 ± 1.55	2.82 ± 1.76	2.59 ± 1.92	F = 29.6 ; p < .0001
Future prospects	2.92 ± 1.60	3.64 ± 1.29	2.22 ± 1.49	2.43 ± 1.66	F = 43.5; p < .0001
Health situation	2.45 ± 1.62	3.20 ± 1.49	1.89 ± 1.39	1.77 ± 1.47	F = 50.3; p < .0001
Abilities to deal with Daily life activities	3.07 ± 1.62	4.03 ± 1.33	2.29 ± 1.26	2.24 ± 1.47	F = 94.8; p < .0001

*Among the group of 145 patients with psychiatric disorders, 23% had obsessive-compulsive disorders (n = 34; F42), 19% borderline disorders (n = 27; F60.31), 19% alcohol addiction (n = 27; F10), 7% other addictive disorders (n = 10; F20), 8% bipolar disorders (n = 12; F31), and various other diseases with < 5% each.

**All scales had a normal distribution, while, typical for secular societies, the low engagement in Religious practices and Spiritual (mind-body) practices resulted in a left screwed distribution.

^§^51% had relapsing remitting MS course, 25% progressive relapsing MS, and 23% chronic progressive MS; mean duration of disease = 10.7 ± 8.3 years; mean “Expanded Disability Status Scale” (EDSS; ranging from 0 to 10) score = 3.7 ± 1.8 (indicating moderate disability).

### Patients

We focused on people living with multiple sclerosis (MS) and psychiatric diagnoses (disease specific details are given in Table 1). Outpatients with MS were consecutively recruited from four specialized hospitals, i.e., Department of Neurology and Palliative Care, Köln-Merheim Hospital, Cologne; Department of Neurology, Communal Hospital Herdecke, Herdecke; Neurological Hospital, Clinic of Lüdenscheid, Lüdenscheid; and Augustahospital, Isselburg-Anholt. Patients with psychiatric disorders were recruited in the Department of Psychiatry and Psychotherapy of the University Clinic Freiburg, Freiburg.

All individuals were informed about the purpose of the respective study, were assured of confidentiality, and were asked to provide informed consent. Ethical approval for the study enrolling patients with MS was obtained by the IRB of Witten/Herdecke University (#21/2012). For the study enrolling psychiatric inpatients, ethical approval was granted by the IRB of the University of Freiburg (#7/10).

### Measures

#### Frequency of engagement in spiritual activities

Spirituality is often regarded as a broader and more individual concept than religiosity, particularly in contrast to formal (institutional) religiosity. Although there is a debate whether or not it is useful to distinguish between the concepts “spirituality” and “religiosity” [26], it might be appropriate for research purposes. Particularly in Western societies, it is important to differentiate between formal religiosity, transcendent experience, existential search for meaning, secular humanism, etc. [27].

To differentiate between various forms of specific spiritual practices, we used the SpREUK-P questionnaire (SpREUK is the German language acronym of “Spirituality/Religiosity and Coping”) [23,26]. The generic instrument was designed to measure engagement in organized and private religious, spiritual, existential and philosophical practices. In its shortened 17-item version, it differentiates between 5 factors [23]:

• *Religious practices* (4 items; alpha = .82; i.e., praying, church attendance, religious events, religious symbols);

• *Humanistic practices* (4 items; alpha = .79; i.e., help others, consider their needs, do good, connectedness etc.);

• *Existential practices* (3 items; alpha = .77; i.e., meaning in life, self-realization, getting insight);

• *Gratitude/Awe* (3 items; alpha = .77; i.e., feeling of great gratitude, feelings of wondering awe, experienced and valued beauty),

• *Spiritual (mind-body) practices* (3 items; alpha = .72; i.e., meditation, rituals, working on a mind-body discipline [i.e., yoga, qigong, mindfulness etc.]),

The items of the SpREUK-P are scored on a 4-point scale (0 - never; 1 - seldom; 2 - often; 3 - regularly). The scores can be adjusted to a 100% scale (transformed scale score), which reflects the degree of engagement in the distinct forms of spiritual/religious practice (“engagement scores”). Scores > 50% indicate higher engagement, while scores < 50% indicate rare engagement.

The mean scores of the respective SpREUK-P subscales were calculated only when 2/3 or 3/4 items of the respective scales were answered. Thus, we did not calculate the mean scores of the scale *Existential practices* in 4 cases, *Spiritual (mind-body) practices* in 9 cases, *Humanistic practices* in 12 cases, and *Religious practices* in 14 cases.

#### Gratitude/Awe

With respect to the research topic, we used the three items of SpREUK-P’s *Gratitude/Awe* subscale as single items (Gratitude, Awe, and Experienced Beauty) and the mean score of the subscale. These items measure a personal disposition rather than emotions that arise when people receive specific aid or gifts (in terms of situational gratitude). Thus, these target items do not refer to specific help or benefactors; rather, they reflect a specific, more generalized attitude in life. This is in line with the conceptualization of Wood et al. [14]. They differentiated between eight aspects of gratitude: (1) experience of grateful affect, (2) appreciation of other people, (3) focus on what a person has, (4) feelings of awe when encountering beauty, (4) behaviors to express gratitude, (5) the positive in the present moment, (6) appreciation for the understanding that life is short, (7) a focus on the positive in the present moment, and (8) positive social comparisons [14].

#### Spiritual/religious self-categorization

According to their responses to the items of the SpREUK-15 questionnaire (item f2.6: “To my mind I am a religious individual” = R; item f1.1: “To my mind I am a spiritual individual” = S), the patients were categorized as religious but not spiritual (R + S-), as not religious but spiritual (R-S+), as both religious and spiritual (R + S+), or as neither religious nor spiritual (R-S-) [25]. The respective items were scored on a 5-point scale from disagreement to agreement (0 - does not apply at all; 1 - does not truly apply; 2 - don’t know (neither yes nor no); 3 - applies quite a bit; 4 - applies very much). Scores 3 and 4 were used as statements of “agreement” (R + or S+). To avoid internal conflicts, we did not provide information on how a religious or a spiritual individual should be defined.

Due to missing data, we were unable to calculate the respective self-categorizations in 31 cases.

#### Life satisfaction

Life satisfaction was measured using the Brief Multidimensional Life Satisfaction Scale (BMLSS) [28], which uses items of Huebner’s Brief Multidimensional Students’ Life Satisfaction Scale [29,30] and was tested among adults [28]. The eight items of the BMLSS address intrinsic (*Myself, Life in general*), social (*Friendships, Family life*), external (*Workplace situation, Where I live*) and prospective (*Financial situation, Future prospects*) dimensions of life satisfaction. The internal consistency of the instrument was good (Cronbach’s alpha = .87) [28]. We added two further health-related variables, i.e. satisfaction with health situation and satisfaction with abilities to deal with daily life activities. Each of these 10 items was introduced by the phrase “I would describe my level of satisfaction as …”, and scored on a 7-point scale from dissatisfaction to satisfaction (0 – terrible; 1 – unhappy; 2 – mostly dissatisfied; 3 – mixed (about equally satisfied and dissatisfied); 4 – mostly satisfied; 5 – pleased; 6 – delighted). The BMLSS-10 sum score corresponds to a 100% level (“delighted”).

To calculate the BMLSS mean scores, 2 missing items were tolerated. Thus, we had no data on life satisfaction in 7 cases.

### Statistical analyses

Descriptive statistics as well as analyses of variance, first order correlations and regression analyses (enter method) were computed with SPSS 20.0.

We judged a p < .05 as significant. For correlation analyses, we chose a significance level p < .001. With respect to classifying the strength of the observed correlations, we regarded r > .5 as a strong correlation, r between .3 and .5 as a moderate correlation, r between .2 and .3 as a weak correlation, and r < .2 as no or a negligible correlation.

## Results

In the following paragraphs, we will first describe the sample, particularly with respect to patients’ spiritual/religious self-categorization and life satisfaction. We will then present their perception of the test variables *Gratitude*, *Awe*, and *Experienced Beauty*, followed by correlation and regression analysis to assess which variables may have an influence on these emotions/perceptions.

### Patients

Among the 461 enrolled individuals, 46% had MS, 22% depressive disorders, and 32% psychotic and other psychiatric disorders (among them, 23% obsessive-compulsive disorders, 19% borderline disorders, 19% alcohol addiction, 7% other addictive disorders, 8% bipolar disorders, and various other diseases with < 5% each). Patients with acute psychotic episodes (neither acute major depression nor other kinds of psychotic episodes), however, were not included. Patients’ mean age was 41 ± 13 years; 68% were women and 32% men (Table 1). All further socio-demographic and disease-related details are presented in Table 1.

Half (50%) of the patients regarded themselves as neither religious nor spiritual (R-S-), 36% as religious (R + S + or R + S-), and 15% as spiritual but not religious (R-S+) (Table 1). The relative proportion of these SpR categories did not differ significantly with respect to the disease groups (Table 1). However, patients with MS had the lowest engagement in *Existential practices* and *Religious practices*. All other forms of spiritual practices did not differ significantly with respect to the underlying disease (Table 1).

The mean life satisfaction score was 55 ± 22 (Table 1), which indicates moderate life satisfaction (compared to healthy individuals who typically score 75 to 80). In our sample, individuals with MS had significantly higher life satisfaction scores than patients with psychiatric disorders (Table 1). As shown in Table 1, with the exception of satisfaction with *Friendships* and *Where I live*, all other life satisfaction sub-dimensions of the patients with psychiatric disorders had scores <3 (indicating dissatisfaction with life). In contrast, patients with MS had scores >3 (indicating satisfaction with life). In particular, MS patients were most satisfied with *Family life*, *Friendships*, *Where I live*, and *Life in general*. The strongest variance was observed with respect to satisfaction with *Life in general* and *Satisfaction with myself* (Table 1). In the entire sample, life satisfaction sum scores did not differ significantly with respect to patients’ spiritual/religious self-categorization (F = 1.2; n.s.).

### Gratitude/Awe in the sample

In the entire sample, 23% never, 43% rarely, 24% often, and 10% frequently experienced *Gratitude*. In contrast, 41% never, 37% rarely, 17% often, and 6% frequently experienced *Awe. Beauty in Life* was never experienced by 8% of the sample, and 28% rarely, 46% often, and 18% frequently experienced it.

*Gratitude* and *Beauty in Life* were experienced significantly more often by women than men (Table 2). Yet, age, family status and educational level had no significant influence on these variables (data not shown). Patients with depressive disorders experienced *Beauty* significantly less frequently than other patients, but no significant difference was found in *Gratitude* or *Awe* scores. Detail analyses showed that patients with MS and those with psychotic and other disorders (yet not depressive ones) differed significantly (p = .024) in regard to their experience of *Beauty*. MS patients reported slightly higher scores (data not shown). Spiritual/religious self-categorization had the strongest impact on these three variables (Table 2). For *Gratitude* and *Awe,* R-S- patients had the lowest scores, while R + S + had the highest scores. For the experience of *Beauty*, R + S + and R-S + showed the highest scores.

**Table 2:** M**ean scores of gratitude, awe, beauty, and gratitude/awe sum score stratified by gender disease and spiritual/religious self-categorization**

		**Experience of**			**Gratitude/awe Sum score (SpREUK-P; 0–100)**
		**Gratitude (0–1)**	**Awe (0–1)**	**Beauty in life (0–1)**	
**Entire sample**	Mean	1.21	.87	1.74	42.39
	SD	.91	.89	.84	23.78
**Gender**					
Female	Mean	1.29	.92	1.80	44.66
	SD	.92	.90	.85	23.97
Male	Mean	1.01	.78	1.59	37.57
	SD	.87	.85	.80	22.35
F value		**9.2**	2.2	**6.0**	**8.8**
p value		**.003**	n.s.	**.015**	**.003**
**Diseases**					
Psychotic disorders	Mean	1.20	.92	1.72	42.56
	SD	.96	.89	.87	25.26
Depressive disorders	Mean	1.11	.94	1.41	38.37
	SD	.92	.95	.77	24.61
Multiple sclerosis	Mean	1.26	.80	1.92	44.32
	SD	.88	.85	.79	22.06
F value		1.0	1.1	**13.6**	2.2
p value		n.s.	n.s.	**<.0001**	n.s.
**SpR self-categorization**					
R+S+	Mean	1.87	1.43	1.98	59.02
	SD	.83	.92	.75	23.74
R+S-	Mean	1.44	.98	1.84	47.27
	SD	.88	.84	.82	23.64
R-S+	Mean	1.32	1.06	2.05	49.51
	SD	.90	.99	.75	24.16
R-S-	Mean	.88	.64	1.52	33.62
	SD	.81	.78	.84	20.09
F value		**25.8**	**15.5**	**10.7**	**26.6**
p value		**<.0001**	**<.0001**	**<.0001**	**<.0001**

Significant differences were highlighted (bold).

### Correlation and regression analyses

Correlation analyses revealed that *Gratitude/Awe* was moderately associated with engagement in various forms of spiritual practices (Table 3). Specifically, *Religious practices* correlated most strongly with *Gratitude* and *Awe. Existential practices* were strongly correlated with *Awe. Humanistic practice* was most strongly associated with *Beauty* and *Gratitude*, and *Spiritual (mind-body) practices* were related to *Awe* (Table 3).

**Table 3:** Correlation between gratitude, awe, experienced beauty, and engagement in spiritual practices and life satisfaction

	**Gratitude**	**Awe**	**Experienced beauty**	**Gratitude/Awe sum score** (SpREUK-P)
Gratitude	1	.591^**^	.509^**^	.876^**^
Awe		1	.304^**^	.793^**^
Experienced beauty			1	.742^**^
**Engagement in spiritual practices (SpREUK-P)**				
Existential practices	.224^**^	.296^**^	.204^**^	.307^**^
Humanistic practices	.351^**^	.269^**^	.379^**^	.413^**^
Religious practices	.457^**^	.459^**^	.250^**^	.483^**^
Spiritual mind-body practices	.305^**^	.350^**^	.302^**^	.396^**^
**Life satisfaction (BMLSS)**	.154^**^	-.047	.350^**^	.186^**^

**p < .01 (Pearson; 2-tailed).

Moderate and strong correlations (r) were highlighted (bold).

In contrast to our hypothesis, *Gratitude/Awe* (SpREUK-P subscale) cannot explain any relevant variance in life satisfaction (R^2^ = .04; Beta = .19; T = 4.0, p = 0.001). Regression analyses (enter method) revealed that *Gratitude/Awe* can be predicted best by *Religious practices*, followed by other forms of spiritual practices and life satisfaction. Female gender is a weak predictor of *Gratitude/Awe*, but the underlying disease and the lack of a SpR attitude had no significant influence (Table 4). These variables explain 42% of *Gratitude/Awe*’s variance. Because the regression coefficients may be compromised by collinearity, we checked the variance inflation factor (VIF) as an indicator for collinearity. A VIF higher than 10 is indicative for high collinearity. Results suggested that collinearity was not a problem in the respective models.

**Table 4:** Predictors of gratitude/awe (regression analysis; enter method)

**Dependent variable: Gratitude/Awe sum score (SpREUK-P subscale)**		**Beta**	**T**	**p**	**Collinearity statistics**	
					**Tolerance**	VIF*
R^2^ = .42	(constant)		0.02	.982		
	Religious practices	.29	6.17	.000	.66	1.51
	Humanistic practices	.24	5.71	.000	.86	1.16
	Spiritual mind-body practices	.16	3.56	.000	.72	1.39
	Existential practices	.13	2.91	.004	.70	1.43
	Life satisfaction (BMLSS)	.22	4.51	.000	.65	1.55
	Male gender	-.08	-2.07	.039	.93	1.08
	Psychiatric patients	-.02	-0.40	.689	.59	1.70
	Neither religious nor spiritual (R-S-)	-.07	-1.48	.140	.67	1.49

*Variance Inflation Factor.

## Discussion

We intended to analyze whether and how often patients with MS or psychiatric disorders experience feelings of *Gratitude*, wondering *Awe*, and *Beauty* in life. Our data show that Gratitude and Awe were noticed and appreciated by a small group (34% and 23% often or frequently, respectively) of the individuals investigated. In contrast, most participants have experienced and appreciated *Beauty* in life (64% often or frequently). The mean scores of the *Gratitude/Awe* scale, which measures the frequency of these feelings and experiences, are in the lower intermediate range (42.4 ± 23.8). Previously, in a positively selected group of Catholic priests, we observed much higher mean scores of 70.1 ± 21.6 [31]. Another sample of patients with chronic diseases (70% chronic pain diseases), reached mean scores of 50.3 ± 23.7 on the respective scale [32].

With respect to our assumption that the perceptions of *Gratitude*, and experience of *Beauty* in life may differ according to gender, we indeed observed lower scores of *Gratitude/Awe* in male patients. This finding is in line with the findings of other studies [33]. This might not mean that men are less capable of experiencing feelings of gratitude or beauty, but, as found by Kashdan et al. [33], women have a higher willingness to express their emotions than men.

Interestingly, there were no significant differences with respect to the underlying disease categories. The only exception was that patients with depressive orders had significantly lower experiences of *Beauty*.

Because one of our hypotheses was that religious/spiritual patients may experience *Gratitude*, *Awe* and *Beauty* more often than non-religious/skeptical persons, we analyzed the respective variables with respect to the spiritual/religious self-categorization of the patients and their engagement in specific forms of spirituality. We found that persons lacking a spiritual/religious attitude (R-S-) had significantly lower *Gratitude/Awe* scores than patients with spiritual/religious attitudes. In line with this finding, *Gratitude/Awe* correlated most strongly with the frequency of engagement in *Religious practices*, but also secular forms of spirituality, particularly *Humanistic practices*. Both activities, religious and humanistic, are associated with relational activities. This can be expressed either through connectedness to a transcendent source (resulting in praying, church attendance, etc.) or connectedness to concrete others (resulting in a helping and caring attitude), and both religious and humanistic practices are primarily active. A previous study also showed that the best predictors of *Gratitude/Awe* were religious trust (in terms of an intrinsic religiosity) [23]. Among the three target items analyzed in this study, feelings of *Gratitude* as well as Awe correlated most strongly with *Religious practices*. Experienced Beauty was most strongly associated with *Humanistic practices* and only weakly with *Religious practices*. Thus, it is the experience of feelings of *Gratitude* and wondering *Awe* more than awareness of *Beauty* in life that might have a positive spiritual/religious connotation.

Keltner and Haidt [9] recommended that research should concentrate on similarities and differences between gratitude and awe. In our study, both were strongly interconnected (r = .59). However, *Awe* was experienced less often than *Gratitude*. Interestingly, significantly more women than men experience *Gratitude*, whereas there were no significant gender differences with respect to *Awe*.

Wood et al. [34] argued that an “ungrateful person is less likely to notice help, and less likely to reciprocate the help, making their benefactor less willing to provide further aid”. In turn, grateful people benefit from “better social relationships, characterized by greater closeness and heightened reciprocal social support”. Patients with reduced experience of gratitude and awe, or low awareness of the (still existing) positive aspects in life (including beauty in nature, situations, and relationships) receive help to focus their attention on these aspects of their life. The persons investigated in this study (particularly those with psychiatric disorders) may benefit from such interventions, which have the potential to increase their life satisfaction and well-being. Gratitude interventions to increase well-being and decrease depressive symptoms might be a meaningful option [14,35].

Previous research has shown that gratitude is strongly related to well-being [14]. However, in this study we measured satisfaction with various dimensions of life concerns and not simply well-being. We observed only a weak association between life satisfaction and *Gratitude*, yet a moderate association with the experience of *Beauty* in life. However, general life satisfaction was among the best predictors of *Gratitude/Awe*. What is measured as life satisfaction or quality of life might differ from what patients and people in general consider as authentic happiness or as lasting personal fulfillment, and from what may constitute elements of personal “well-being”. Fagley [36] found that awe correlated moderately with life satisfaction (r = .35) and only weakly with gratitude (r = .28); their hierarchical multiple regression analysis indicated that after controlling for demographic variables, personality factors and gratitude, a grateful attitude made a “significant unique contribution (11% of the variance, p < .001) to life satisfaction”. In our study, *Gratitude/Awe* explained only a small amount of life satisfaction (4%), and thus it should be regarded as an independent dimension. Our findings emphasize the fact that more frequent experiences of *Gratitude/Awe* will not necessarily result in higher life satisfaction with all its usually scored aspects, both objectively and subjectively. Rather, it may indicate an ability of patients with a spiritual/religious attitude to “look deeper” and to appreciate life as such – a trait or attitude which can be predicted best by an engagement in *Religious practices* and *Humanistic practices*.

Findings of Diener et al. [37] showed that the association between religiosity and higher subjective well-being is mediated by social support, feeling respected, and meaning in life. Interestingly, the prevalence of religiousness was dependent on the characteristics of the society and underlying difficulties of life conditions. They found that in societies with more favorable circumstances, individuals’ religiosity was lower, and the level of well-being was similar among religious and non-religious individuals [37]. Also, in the German patients investigated in this study, the percentage of religious individuals was low (65% non-religious), and their relatively low life satisfaction scores did not differ with respect to the underlying spiritual/religious self-perception. This means religious/spiritual patients from our sample are not more satisfied with their life concerns (as measured by life satisfaction scales) than non-religious patients.

Although religious self-perception appears to have no significant influence on life satisfaction, a study among healthy individuals showed that higher levels of trait gratitude are associated with “more positive beneficial appraisals” [38]. Because previous studies found that positive spirituality/religiosity was significantly associated with positive appraisals of chronic illness [32], one may suggest that patients in our sample also draw on spiritual/religious resources to cope with chronic illness (in terms of finding meaning and hope) and to value the moments of beauty and show gratitude for the positive aspects in life despite illness. This attitude might be a dispositional trait, which can be developed (i.e., gratitude interventions (reviewed by [14]). Wood et al. [14] stated that there might be a “higher order grateful personality” that exists beyond particular aspects of gratitude and may represent a “life orientation towards the positive”, involving a “worldview towards noticing and appreciating the positive in life”.

In cancer patients, Strack et al. [20] reported that the “cognitive reappraisal of emotion, gratitude finding, and openness to experience” was associated with post-traumatic growth in patients’. In our study, we focused on two groups of patients with primarily non-fatal, chronic diseases, who have to find strategies to adapt to their often recurring symptoms. In this sample of non-cancer patients, we have evidence of relatively low life satisfaction, particularly in the patients with psychiatric disorders. Detailed analyses showed that *Gratitude* and *Awe* are not experienced or noticed very often. However, the participants have still experienced and valued *Beauty* in life. One may hypothesize that patients lack reasons to be grateful because of their frustrations with the disease and associated dissatisfaction with life; or they give less attention to issues other than the symptoms of their illness. However, it seems that religious individuals are able to value other aspects in their life despite the disease – though they need not necessarily be more satisfied with their life in the *usual* understanding of “life satisfaction”.

### Limitations

This study was not designed to specifically measure gratitude as a short-term emotion. Rather, it is measured here as a disposition of gratefulness and an attention to beauty in life as a specific aspect of spiritual activities and experiences. Therefore, the three items of the *Gratitude/Awe* scale measure a specific aspect of patients’ experience of feelings of *Gratitude*, wondering *Awe* and perception of *Beauty* in life. These can be observed even in patients lacking any interest in spiritual or religious issues.

Further, the study was cross-sectional, and thus we cannot draw conclusions on the directions of causality in the observed associations. Thus, longitudinal studies are strongly needed. Moreover, the perceptions of patients with psychiatric disorders may be significantly different during their “silent” and asymptomatic phases. Similarly, the respective scores might also be lower in patients with MS during acute or relapsing phases of disease. In addition, we have no data on whether or not analyzed patients with MS had frontal lobe lesions or ideation/thought disturbances. Such neurological affections may have an impact on states of consciousness, ideations, and self-reflection. Further research might focus on such altering impacts which have not been pursued by our investigation. Moreover, it would be desirable that future research compares also non-clinical populations and different clinic al populations with regard to the variables at stake.

Nevertheless, the strength of this cross-sectional study is its focus on participants with primarily non-fatal diseases. Instead of choosing a sample of relatively young and healthy students and confronting them with hypothetical situations to induce feelings of gratitude, we chose a sample of patients that has had to deal with the ups and downs of chronic disease. However, we do not know any specific details about our patients’ concrete feelings of gratitude and awe or their specific causes.

Of course there are some additional findings on the putative association between patients’ spirituality and further health-related and psychological measures. Among patients with MS for example, their engagement in religious practices was only marginally associated with negative mood states such as grief, despair or tiredness (r < .20; p < .05), while there were no significant correlations with cognitive or motoric MS related fatigue (r < .10; n.s.) (Wirth et al., in preparation). These and other details will be topic of independent papers of the Freiburg group on patients with psychiatric diseases (Reiser et al., in preparation) and the Herdecke group on patients with MS (Wirth et al., in preparation).

## Conclusions and outlook

*Gratitude/Awe,* as measured in this study, could be regarded as a “life orientation towards noticing and appreciating the positive in life” [14]. This was significantly associated with specific spiritual/religious attitudes. Positive spirituality/religiosity seems to be a source of gratitude and appreciation of life in all its dimensions including “light and shadows”. In contrast, a non-religious/non-spiritual (R-S-) self-perception was associated with lower abilities or perceptions of *Gratitude* and *Awe* in life. Diener et al. [37] confirmed an association between religiosity and higher subjective well-being, which is mediated by social support, feeling respected, and meaning in life. Thus, patients need access to available resources and strategies. However, not all patients are able to recognize and/or value such internal and external resources. It seems that the personality prior to illness has an influence on the ways individuals will adapt and cope. Moreover, a nationally representative, longitudinal study performed by the University of Manchester (using a shortened version of the “Big Five Inventory”), found that agreeable individuals adapt more easily and fully to health afflictions than disagreeable individuals [39]. Spirituality/religiosity might also be one aspect that allows patients to cope more easily. Medical professionals should be aware of such personality differences because individuals lacking such resources for coping may need additional and specific support to adapt.

Further research should address the relationship between *Gratitude/Awe* as a dimension of spirituality/religiosity along with personality traits like the “Big Five” (openness, conscientiousness, extraversion, agreeableness, and neuroticism). Is agreeableness a predisposition for, or rather a consequence of gratitude – or are they mutually reinforcing?

Boyce and Wood [39] have shown a critical predictive role of agreeableness prior to disability for the adaptation to and coping with disability as well as for the recovery of lost life satisfaction within four years. Whether the same might also be true for patients with neurological and/or psychiatric diseases remains to be shown.

## Competing interests

The study was not financed by any organization; the authors did not receive financial support by organizations, companies etc. which could have influenced the interpretation of data.

## Authors’ contributions

AB conceived the study, performed statistical analysis and drafted the manuscript. AGW, AZ, FR, and KB participated in the conception and design of the study. AZ, KH, KG, MH, and SS helped to recruit the patients. KB contributed to drafting the manuscript. All authors read and approved the final manuscript.
